# Hemiplegias

**Published:** 1903-02-21

**Authors:** 


### Hospital Clinics and Medical Progress.

### HEMIPLEGIAS.

When we note the large number of people killed or changed into miserable wrecks by the common forms of hemiplegic paralyses it is evident that few things are more worthy of study than the means of preventing and treating these so-called "strokes." The subject of granular kidney would in many cases live almost as long and work as well as a healthy person if cerebral hæmorrhage did not come on with its resulting hemiplegia. The aged or the convalescent after acute illnesses are cut off because we do not foresee and prevent thrombosis. The sufferers from endocarditis are crippled by embolic lesions, and the syphilitic die from changes in the cerebral vessels, not to mention hemiplegias from rare causes such as cerebral tumours. Roughly speaking, the three great causes of these paralyses are hæmorrhages, thrombi and emboli. How far are they preventable, and how far are the lesions permanent?

The hæmorrhages are due to vascular degenerations in senile decay, in renal cirrhosis, in gouty, alcoholic, and atheromatous states; occasionally to direct injury, syphilitic lesions, purpura and other blood diseases. Pathologically they are most commonly due to fibrotic and atheromatous changes, miliary aneurisms and syphilitic changes. Possibly all these are varieties or stages of fibroid degeneration due to the action of different toxins on the vessel walls. Vessels in these conditions very frequently, but not universally, give way, and on the other hand a few cases of hæmorrhage do occur with perfectly healthy vessels and normal hearts.

A hypertrophied heart of course increases the risk of diseased vessels failing; but provided the vasodilator mechanism is perfect it is not clear that any rise of pressure could take place, even in diseased vessels, since there are always sufficient healthy vessels to carry off the blood stream if all the dilatation possible takes place.

If this position be granted, the possibility of keeping down dangerous pressure depends on how far we can overcome vaso-contraction, and not on preventing cardiac hypertrophy. Indeed, such hypertrophy will soon disappear of itself if the opposing force is removed. How far, then, can we prevent hæmorrhages? In two ways, first by maintaining vascular dilatability, and, secondly, to guard against the effects of a sudden accidental rise we must endeavour to avoid degeneration of the vessel walls.

As to the first, then, we find that in the diseases such as renal cirrhosis where high pressure occurs, that much can be done by careful treatment, continued for months and years. Iodide of sodium may be given for many weeks at a time, again and again. The nitrites may be used when a temporary rise of pressure occurs, in small, frequently repeated, but increasing doses. Regular periodical saline purgatives will aid the deficient action of the kidneys, and of themselves lower the pressure directly. If the kidney epithelium can be stimulated to the excretion of solids rather than of water, the cause of vaso-contraction may be removed. Similarly, if we can prevent the absorption of the poisons of gout, alcohol, and lead, vaso-contraction may be prevented. Elimination is difficult with defective kidneys, but in gout the formation of uric acid may be at times prevented by individual dieting, as well as by the use of colchicum and guiacum. In France we find various diseases treated by placing the patient on a special diet, say milk, every third or fourth week for long periods, and something of the kind may be usefully tried in renal cirrhosis if the patient can be watched. Of course meat soups, extracts, and alcohol should be reduced to a minimum, even in the rest of each month. In every possible way we must strive for long periods to prevent dangerous high pressures, since we can never tell when a sudden accident may strike down our patient and do far more harm than the original disease.

As to preventing vascular degeneration we are ignorant of any effective remedy, except so far as we can treat the original disease. Rumpf has recommended in cases of atheroma a lime-free diet consisting of meat, fruit, and rice, and the administration of lactic acid which is worth considering, and, of course, in syphilis we must not fail to use vigorously specific remedies at once and for long periods.

When hæmorrhage has taken place the immediate treatment is well known. Purgation to lower pressure and increase the viscosity of the blood, chloride of calcium to aid coagulation if the clotting time is short, cold to the head, and absolute rest are among the chief points. Bleeding should be employed whenever there is a strong hard pulse and cyanosis. Indeed, as a preventative measure it is probable that we have gone too far in its disuse. The value in uræmic states of venesection, coupled with saline injections, is evidence of its utility. The diagnosis of a hæmorrhage from a vascular occlusion is easy in most severe cases, but difficult in mild ones. The state of the heart and pulse, the deep progressive coma, the fall and subsequent rise of temperature may or may not enable us to decide with certainty. Yet a decision is important since the depressing treatment needed in hæmorrhage is harmful in thrombosis.

The common causes of emboli, according to J. Taylor, are second attacks of endocarditis, especially in mitral stenosis and occasionally in aortic disease; clots in the left auricle after a failure of compensation, especially when the blood clots very freely, as in pregnancy, lactation, and rheumatism; aneurysms and atheroma; and certain lung diseases, such as an old empyæma and various septic conditions. The paralysis produced is sudden and without premonitory symptoms. Speech is often affected, for in 60 per cent. of the cases the left side of the brain is injured and the middle cerebral artery is the favourite vessel. Loss of consciousness is common, but the paralysis rarely turns out to be as extensive as it appears at first. In thrombosis extensions may take place two or three weeks later, and in either disease aneurysms or other changes may arise in the occluded vessel.

The detachment of an embolus can hardly be prevented. Sudden exertions in the states mentioned above should be forbidden when possible. Thrombi from which some of these are detached will be discussed below. Septic conditions should be actively treated, and in rheumatic endocarditis alkalies in large doses and iodides, with rest for long periods, are important preventatives.

(*To be Continued.*)

